# The Toxicity of Depleted Uranium

**DOI:** 10.3390/ijerph7010303

**Published:** 2010-01-25

**Authors:** Wayne Briner

**Affiliations:** Department of Psychology, University of Nebraska at Kearney, Kearney, NE 68818, USA; E-Mail: brinerw@unk.edu; Tel.: +1-308-865-8196; Fax: +1-308-865-8980

**Keywords:** depleted uranium, heavy metal, toxicity

## Abstract

Depleted uranium (DU) is an emerging environmental pollutant that is introduced into the environment primarily by military activity. While depleted uranium is less radioactive than natural uranium, it still retains all the chemical toxicity associated with the original element. In large doses the kidney is the target organ for the acute chemical toxicity of this metal, producing potentially lethal tubular necrosis. In contrast, chronic low dose exposure to depleted uranium may not produce a clear and defined set of symptoms. Chronic low-dose, or subacute, exposure to depleted uranium alters the appearance of milestones in developing organisms. Adult animals that were exposed to depleted uranium during development display persistent alterations in behavior, even after cessation of depleted uranium exposure. Adult animals exposed to depleted uranium demonstrate altered behaviors and a variety of alterations to brain chemistry. Despite its reduced level of radioactivity evidence continues to accumulate that depleted uranium, if ingested, may pose a radiologic hazard. The current state of knowledge concerning DU is discussed.

## 1. The Early History of Uranium

Uranium was first described in a scientific manner by the German pharmacist Klaroth who isolated it from “pitchblende”, a waste mining ore at the time. Uranium then began to be used in the manufacture of ceramic and glass vessels as well as paints. Uranium and pitchblende began to receive scientific attention after Madame Marie Curie isolated radium and interest gradually increased with the expansion of radiochemistry and radiation physics. Diseases of miners were the first to be linked to the risks of uranium, even before uranium was the purpose of the mining. Pitchblende, a waste ore in silver, bismuth and cobalt mining, was already linked to lung disease in miners in the mid 1500s. The radioactive dust from these mining operations, as well as radon exposure, produced noteworthy rates of lung cancer in German miners [1].

During and after World War Two uranium mining became a purposeful and concentrated concern. During this time industrial hygiene generally improved and the study of uranium was part of the overall effort to study the sequela of nuclear war which focused on radiation exposure from a variety of elemental isotopes. The chemical toxicity of uranium was de-emphasized.

After the Cold War ended and the threat of widespread nuclear war diminished the threat of exposure to uranium and other similar metals also seemed to diminish. However, with the first Persian Gulf War in 1990−1991 depleted uranium (DU) munitions were introduced into the battlefield in a significant way [2]. Gulf War Syndrome was reported after the war, with DU held as a possible causative agent. Because DU is significantly less radioactive than natural uranium, but has identical chemical behavior of uranium in its natural state, investigators began to reconsider uranium as a chemical toxin. The continued use of DU weapons in the Balkans conflict, the Afghanistan war and the second Persian Gulf War have continued to stimulate research into the toxicity of uranium. When DU was introduced to the world audience nearly two decades ago the number of scientific papers on DU in the National Library of Medicine totaled nine. As of this writing the number of papers on DU has reached nearly 400.

This review will focus primarily on the chemical toxicity of DU in an effort to separate the chemical toxicity of uranium from its radiologic effect and will distinguish between low and high dosage exposures. Natural uranium will be mentioned when there are gaps in the DU literature or to emphasize certain points. This review will mention the radiologic effects of DU only for the sake of completeness, and the reader is referred to other sources for an authoritative discussion [3].

## 2. Exposure and Pharmacokinetics

The vast majority of DU that is free in the environment, and a possible source of human exposure, comes from the use of DU munitions. DU munitions are favored as a means to destroy enemy armor. Once they are fired and impact a target DU munitions form small particulate dust and leave behind larger fragments [4]. Potential exposure to DU comes from entry into wounds, inhalation of dust, contact with skin, and the entry of DU into food and water sources. There is general agreement that inhalation of dust is probably the most significant route of exposure [2]. Natural and depleted uranium shows little mobility in the soil but the particles can be re-suspended in the air and inhaled. Skin contact results in vanishingly small amounts of absorption. Water and food intake may play a role in areas with the right soil chemistry and rainfall, with a recent report indicating more mobility in soil than previously appreciated [5]. However, there is little data in the literature and definitive statements are not possible. For these reasons dust inhalation is considered the most important route of exposure.

When DU munitions strike their target a cloud of DU particles, ranging from 0.2−15 microns in diameter and consisting of a variety of oxides, is produced [4,6]. When inhaled these particles are either trapped in the oropharynx, where they are eventually swallowed, or they reach the lower airways where they are subject to alveolar absorption. Alveolar absorption appears to occur in two phases. There appears to be an early rapid phase which results in peak plasma levels and then a decline followed by a prolonged period of steady absorption [7]. It is unclear what accounts for this biphasic pattern. It could be due to the heterogeneous chemistry of DU particles with some compounds simply being more soluble than others. It could be due to the various sizes of inhaled particulates with those of a greater surface area to volume ratio dissolving quickly leaving behind those that dissolve more slowly. It may also be due to an inflammatory response of the lung tissue that begins to retard absorption after a few days [7]. Whatever the mechanism, inhaled DU appears to have a pulmonary half-life of about 4 years [8].

Once absorbed uranium is widely distributed throughout the body. Bone acts as a reservoir for the metal and once environmental uranium exposure has stopped it will be released from the bone for months or years to come. Elimination is principally through the urine making the kidney particularly vulnerable to damage from uranium and high levels of uranium accumulate in renal tissue [9]. At higher doses of uranium kidney damage is the primary concern and the most immediate threat to patient health and survival. However, recent work has shown that chronic low dose DU exposure (12 months in the rat) can produce subtle pathologic changes in the kidney along with blood chemistry changes suggesting renal dysfunction [10] with accompanying anemia due to aberration of the kidneys erythropoitic function [11].

## 3. Pharmacodynamics

DU crosses the blood brain barrier and accumulates in the brain and preferentially in particular brain regions. Specifically, the hippocampus and striatum accumulate DU more readily than cerebellum and cortex, at least with oral exposure [12]. In contrast, when using a dust exposure protocol, the accumulation of DU in the CNS occurs within the olfactory bulb, hippocampus, cortex, and cerebellum demonstrating increasing concentrations of DU, in that order [13].

An early study [14] produced tremors in rats given high doses of uranium. That finding, in concert with the activity of uranium at the neuromuscular end-plate [15] suggests that U competes with calcium at the synapse. DU is active at the estrogen receptor [16], the vitamin D receptor [17], and the retinoid receptor [17]. DU appears to alter acetylcholine and serotonin activity [18], although these findings are not always consistent [12]. We have recently shown DU to effect brain norepinephrine and epinephrine levels [19]. However, the effect of DU on brain dopamine appears complex and related to both length of exposure and dose [19]. Interestingly, a variety of physiologic changes have been reported in the intestine of rats being administered DU in drinking water including altered histamine, prostaglandin and NO activity [20]. Our laboratory has consistently shown that DU exposure increases brain lipid peroxidation, a finding replicated by others [21–23]. Brain lipid peroxidation parallels behavioral changes in rats and mice exposed to DU [24–26]. Changes in brain lipids may be partially explained by altered gene expression for cholesterol metabolism [27]. Uranium increases the levels of a variety of proteins involved in metal metabolism including divalent metal transporter 1, ferritin, and ceruloplasmin [28].

DU not only accumulates in the CNS but also has physiologic activity there. Specifically, DU inhibits spike formation in the hippocampus of rats [29]. Research has also demonstrated that DU alters the electroencephalographic architecture of the EEG in free moving rats with accompanying changes in the sleep wake cycle and REM sleep [30,31]. DU exposure effects the behavior of rats in the open-field and the Y maze implying that DU has neurophysiologic effects [12]. This laboratory has also demonstrated a number of behavioral effects associate with DU exposure, such as altered development and maze behaviors [26,32]. However, others have found that neither sleep wake cycles or spatial behavior were altered by either DU or enriched uranium exposure [33].

## 4. Acute High Dose Exposure

Acute exposure to large doses of uranium occurs rarely and is not the focus of the paper, but is mentioned for the sake of completeness. Large dose acute exposure to uranium would probably be the result of an industrial or laboratory accident. Oxides, common salts of uranium, and uranium metal offer little risk unless ingested or inhaled in some manner. Other forms of uranium (e.g., uranium hexafluoride) are a direct chemical hazard due to their corrosive nature. Regardless of the form if large doses are ingested or inhaled acute renal failure due to tubular necrosis is the result. The LD_50_ of uranium for humans has been calculated to be about 14mg/kg, depending on the chemical form [34]. Depending on the dose the kidney may recover spontaneously or with dialysis. Rats exposed to uranyl acetate (1 mg/kg injected) demonstrated tubular necrosis and changes in blood chemistry reflecting renal compromise. After 30 days regeneration had taken place, however, cortical scarring and interstitial nephritis persisted [35]. Treatment of uranium intoxication involves removal of the source of exposure and supportive treatment. Chelation strategies are of questionable value because these agents require that the uranium-chelation complex be secreted by the kidney, which is already compromised. However, the compound CBMIDA has shown promise by reducing uranium burden without causing renal damage and may be administered orally [36].

## 5. Chronic Low Dose Exposure

The remainder of this paper will focus on long duration exposure to low dosages of uranium. This concept implies that the exposure is not sufficient to produce the classic signs of toxicity, in this case renal failure, but would produce other detrimental effects. It is postulated that low level exposure to uranium for extended periods would produce an important low level or “subclinical” illness. We know this to be the case for lead exposure, where long term exposure to small amounts of lead can produce subtle neurodevelopmental defects, hypertension and increased rates of carcinogenesis. Uranium, however, has not been as well studied and population based examinations of the effects of DU exposure have not been done. Despite these shortcomings the research evidence to date is providing us with important clues to the effects of sub-acute DU exposure.

### 5.1. Neurodevelopmental Effects

To date, all our knowledge about the potential neurodevelopmental effects of uranium comes from experimental work on animals. Doses of uranium given to pregnant animals that are not sufficient to produce renal damage can produce small litters, smaller offspring size, increased offspring mortality and skeletal abnormalities. Human studies of the teratogenic effects of DU, while suggestive, suffer from a number of methodological issues [37].

Exposure of developing animals to DU in drinking water results in lower day to day uranium exposure but for a longer period of time. This experimental approach is more likely to parallel long term human dosage and exposure to DU. This type of exposure produces subtle derangements of behavior. In rodents exposure to DU during development actually accelerates the appearance of a number of behaviors (righting reflex, forelimb placing, grasping, swimming and weight gain) [38]. At face value accelerated development may seem little to be concerned about. However, it is important to ask the question; what neural systems are being sacrificed to produce these accelerated behavioral end-points? This question is partly addressed when looking at the behaviors of adult rodents that were exposed to DU during development. Those animals demonstrate altered behavior on the vocalization, touch response, tail pinch, arousal and reactivity tests of a standardized neurodevelopmental test battery. Adult animals exposed to DU during development also performed worse on a test of working memory and had smaller brain weights (as a percentage of body weight) [32]. There does not appear to be any data on the neurodevelopmental consequences of DU exposure in humans or any other primate species.

### 5.2. Neuropsychological Effects

Much of our knowledge of the human effects of DU comes from studies examining Gulf War veterans exposed to DU. Scientific studies directly addressing the health effects of DU on humans are few. The studies that have been done use soldiers and are limited by small sample sizes and the heterogeneous nature of the study groups. Studies on Gulf War veterans have found elevated DU levels in urine linked to embedded DU fragments [39]. DU urinary excretion has also been linked to high serum prolactin levels and neurocognitive performance [40]. To date we have not been able to find any long term studies addressing neuropsychological effects of DU exposure.

Adult animals exposed to DU show subtle but important changes in behavior, including increased activity in a test apparatus and impaired working memory. These behavioral changes correlate with DU mediated lipid peroxidation seen in the brain [26]. Overall, exposure to DU appears to impair the animals ability to modulate its responses to novel environments.

### 5.3. Other Effects

Whether or not DU causes cancer is a popular question in the public media and controversial in the scientific literature. While the radioactivity of DU is low, it is not absent. It has been pointed out [41] that if even a little as 1−2% of the 300 tons of DU used in the Gulf War were converted to respirable dust it would produce three to six million grams of DU dust. Using the figures provided by Durakovic this would release 1.16 million to 2.32 million Ci of radiation, a measure that would exceed the New York state safety levels for monthly release of 150Ci by a factor of 7,733 to 15,467. However, it is unlikely that this amount would be inhaled or ingested by a population, most of it would probably end up in the soil or diluted by the wind. Nonetheless, these figures suggest that it may not be prudent to completely ignore radiation risks from DU.

Human studies examining the carcinogenic potential of DU are limited but suggestive. Evidence of potential carcinogenic effects include suggestions of an increase in cervical carcinomas in Yugoslavia [42,43] and increases in micronuclei formation in subjects from the Bosnia/Herzegovina region [44]. There are also indications of hypoxanthine-guanine phosphoribosyl transferase (HPRT) mutation in some Gulf War veterans [40] as well as chromosomal aberrations in a German study group [45]. Two studies have found suggestive chromosomal aberrations in workers exposed to DU [46,47].

Animal studies, while small in number, are also suggestive of some carcinogenic potential. DU has been shown to produce altered gene expression *in vitro* [48] as well as producing breakage of DNA strands [49] and carcinogenic mutation of human bronchial tissue in culture [50] and genomic instability of cultured human osteoblasts [51–54]. Genetic changes in mouse macrophages [55] and the production of soft tissue sarcomas in rats [56] has been seen with DU. DU also produces increased urinary mutagenicity of using the Ames test in rats [57]. DNA methylation was seen in a rat model of DU-induced leukemia [58]. A study out of France examined the effect of DU on mouse oocytes and found a reduction in the ‘quality’ of the female gamete [59]. Enriched uranium, but not DU had a significant impact on the testicular function of rats [60].

### 5.4. Treatment of Low-Dose Chronic Exposure

It is difficult to discuss treatment for the exposure to a substance when there is neither a clear definition of what would constitute low dose exposure or clear-cut evidence pointing to an adverse outcome, at least in humans. Yet, our previous experiences with heavy metals would suggest that no benefit can come from exposure to uranium. If a proactive stance were taken to remove the theoretical risk from low-dose DU exposure it would seem sensible that removal of DU from the environment would be the first step. How this would be accomplished is unclear and subject to an economic analysis as to the benefit gained if, at least so far, the risk is theoretical. Once exposed to DU has occurred the next steps to be taken are again unclear. Strategies for other metals, such as lead, include chelation, improved nutrition, antioxidants, and environmental enrichment programs. However, as the state of knowledge currently stands, there is no basis for an informed discussion.

## 6. Conclusions

The question of whether or not long term low-level DU exposure is a hazard to human health is a new one. Up until this point uranium exposure has been mostly limited to workers in the nuclear industry. The use of DU weapons has brought uranium exposure to military personnel and the population at large. Moreover, this problem will persist for some time. Uranium persists in the environment for extended periods and military use of this material will likely continue. Indeed, there is speculation that DU weapons are being developed by other nations and will see an expanded role in future warfare. This brings us to the point where we are currently at, where the potential for DU exposure is increasing but our knowledge of potential health outcomes from exposure falls short. Animal studies suggest that DU can have a negative impact on the brain, kidney, and bones of mature animals. The data also suggests that the developing animal may be at risk and that there may be a greater risk of radiation induced cancers that originally thought. But, all of this data is only suggestive until it can be tied to meaningful human research. We do not even have a reasonable idea of the typical DU intake of people living in regions where DU munitions have been used. This lack of fundamental information makes extrapolation from animal data to humans a strictly academic exercise. The few human studies that are out there are limited to military service personnel who have different exposure profile than we would expect for civilians, or the studies are limited in usefulness by their design and sample type [2].

There is a fundamental need for well designed studies that examine the exposure of citizens to DU, and the potential consequences of that exposure, using appropriate control groups (such as citizens in areas of similar demographics that are not exposed to DU). Such studies would probably benefit by examining populations where the exposure to DU was reasonably heavy. In the situation of war zones however, other chemical contaminant and the disruption of society would complicate such studies. A recent study has examined DU contamination from a DU munitions plant in Colonie New York, a population not exposed to the deprivations of war. This would seems to be an excellent group to study to sort out the effects of DU exposure [61]. The research also needs to be conducted in such a way as to have sufficient power to detect adverse effects. This includes not only good sample characteristics but the use of dependent variables that would reflect the potential consequences of DU exposure. I have previously argued that lead may be a good model for DU, at least until more human data becomes available [2]. DU and lead share a number of physical and biochemical characteristics making it reasonable to look for dependent variables that mirror the effects of lead.
